# Towards a Real-Time Embedded System for Water Monitoring Installed in a Robotic Sailboat

**DOI:** 10.3390/s16081226

**Published:** 2016-08-08

**Authors:** Andouglas Goncalves da Silva Junior, Sarah Thomaz de Lima Sa, Davi Henrique dos Santos, Álvaro Pinto Ferrnandes de Negreiros, João Moreno Vilas Boas de Souza Silva, Justo Emílio Álvarez Jácobo, Luiz Marcos Garcia Gonçalves

**Affiliations:** 1 Departamento de Engenharia de Computação e Automação, Universidade Federal do Rio Grande do Norte, DCA-CT-UFRN, Campus Universitário, Lagoa Nova, CEP 59078-970 Natal, RN, Brazil; andouglasjr@gmail.com (A.G.d.S.J.); sarah@dca.ufrn.br (S.T.d.L.S.); davihenriqueds@dca.ufrn.br (D.H.d.S.); alvaro.negreiros@gmail.com (Á.P.F.d.N.); justo.alvarez@gmail.com (J.E.Á.J.); 2 Instituto Federal de Educação, Ciência e Tecnologia do Rio Grande do Norte, Campus Mossoró, Rua Raimundo Firmino de Oliveira, 400, Conj. Ulrick Graff, CEP 59628-330 Mossoró, RN, Brazil; 3 Diretoria Acadêmica de Gestão e Tecnologia da Informação, Instituto Federal de Educação, Ciência e Tecnologia do Rio Grande do Norte, Av. Sen. Salgado Filho, 1559, Tirol, CEP 59015-000 Natal, RN, Brazil; joao.vilasboas@ifrn.edu.br

**Keywords:** sensors architecture, monitoring system, autonomous robotic sailboat

## Abstract

Problems related to quality (and quantity) of water in natural resources or in artificial reservoirs are frequently arising and are at the center of attention of authorities and governments around the world. Many times the monitoring is not performed in an efficient time frame and a precise manner, whereas the adoption of fast and punctual solutions would undoubtedly improve the water quality and consequently enhance the life of people. To minimize or diminish such kinds of problems, we propose an architecture for sensors installed in a robotic platform, an autonomous sail boat, able to acquire raw data relative to water quality, to process and make them available to people that might be interested in such information. The main contributions are the sensors architecture itself, which uses low cost sensors, with practical experimentation done with a prototype. Results show data collected for points in lakes and rivers in the northeast of Brazil. This embedded system is fixed in the sailboat robot with the intention to facilitate the study of water quality for long endurance missions. This robot can help monitoring water bodies in a more consistent manner. Nonetheless the system can also be used with fixed vases or buoys in strategic points.

## 1. Introduction

Water is an inorganic substance of fundamental importance for the existence of living beings. It is the most abundant constituent of living bodies, acting as a universal solvent dispersing organic and inorganic compounds and is essential to biological and chemical reactions that happens in several solutions. Also, it works as a transporting vehicle that makes the exchange of intracellular and extracellular substances and plays a role of great importance as a reagent in molecular transformations [1,2]. Due to the great importance of water for the planet’s life, it has become the focus of discussion in several forums mainly with the goal of finding solutions for the lack of water and its misuse. It is known that only 3% of the existing water is appropriate for consuming, thus to be shared between animals and humans. This is not only for drinking, it is for all the activities in which clean water is necessary.

Preservation of natural resources has been discussed so far by international organisms (United Nations) with specific forums for water as the Mar del Plata Conference (in 2007). National organisms as the Brazilian CONAMA (Environment National Council) [3] and other nationwide agencies are also working on making norms and laws for helping monitoring the natural resources, worldwide. In Brazil, the ANA (Brazilian National Water Agency) is the institution that oversees and puts into practice the laws regulated by CONAMA, allowing Brazil to comply with the agreements reached at international conferences.

With the increasing of Earth’s population, pollution and contamination of sources of water by human action is another problem that appears. The lack of sanitation and industrial waste dumped into rivers and lakes yet further decreases the amount of clean water available for drinking. Environmental pollution directly affects the volume of water available for drinking and may destroy the ecosystem if the concentrations of these pollutants reach higher levels. Beyond these anthropic actions, increasing in water consumption in planetary scale plus an excluding development models point to the emergence of a crisis that must be quelled through integrated water resources management programs. Thus to analyze and, more specifically, to monitor the water of lakes, rivers, and properly, the sea is of fundamental importance for keeping life in the planet.

Towards this direction, as the first contribution, we propose a new hardware and software architecture of an embedded system, in order to allow having installed sensors in an autonomous sailboat (a robot [4]) for water monitoring in reservoirs, rivers, and lakes, with real-time and on-line operation. As a major contribution, the system as proposed (installed in a sailboat robot) supports long endurance operation thus allowing autonomous monitoring. Another important contribution of this work is the new methodology for monitoring that is currently developed as a prototype, including a mechatronic system for measuring the quality of water repeatedly, making possible a long endurance monitoring. Also, as technical contribution, we provide a way for having the psycho-chemical parameters being shown in real time, making data available over the Internet for bodies managers, companies, research institutes and other interested users to monitoring and mitigation in situations caused by environmental problems. The idea is to allow intervening in the polluted ambient with prevention actions previously to a total degradation of the environment.

We come up with a system that is particularly important for defining strategies to the nowadays experimented water problems cited above and also to mitigation of eventual problems that eventually happened due to misleading in water management. It is important at this time to make a difference between monitoring and diagnosis. Monitoring is the repetitive observation of an area or phenomenon with a frequency that is defined according to the variability and the need of information about the behavior dynamics. In other words, monitoring aims to find variations during time rather than determining absolute values for some variables of interest. In this way, the norms applied to acquisition and analysis of data in laboratories do not apply in this process. Nowadays it is not possible for a system, yet, to analyze indicators that need to be collected and analyzed by way of laboratory equipments. In this sense, we believe that great scientific contribution of this work is the proposal, design and development of a physical system that can be effective applied to long endurance monitoring in autonomous way, rather than only taking a few measures in a given time instant.

We initially discuss some basic knowledge on water analysis including variables, sensors and vehicles used, and then present the state of art pointing the main works done in this subject. Then the sensors architecture used for instancing the values and communication and processing unities are introduced. Finally, explanation about each system module is presented including an analysis of the general functioning of the architecture. Results of data collected in lakes and rivers in the northeast of Brazil are presented to validate the system as a whole.

## 2. Variables, Sensors, and Vehicles Used for Water Analysis

In order to analyze the quality of water in reservoirs, rivers, ponds, and in the sea, variables for measuring physical-chemical parameters are essential for accurate studies and also for the search of solutions to the marine fauna and flora issues. These variables are collected by sensors that are installed in vehicles, or it could be manually taken. In this work, the idea is to embark them so a platform has been devised specifically for this end. Some systems and related theory to all these issues are described in the next.

### 2.1. Health Variables of Water

According to Pivele [5] and Fiorucci [6], some variables are of vital importance for aerobic respiration processes, such as dissolved oxygen, and for maintaining an environment that allows the performance of chemical reactions that are important for life, such as the pH. The analysis of these two parameters is thus of fundamental importance for marine life keeping. Also, it is known that oxygen is a slightly water-soluble gas, varying the solubility of 15 mg·L${}^{-1}$ at 0 to 8 mg·L${}^{-1}$ at 25 ${}^{\circ }$C depending on the pressure (altitude) and dissolved salts [7]. Dissolved oxygen is thus the main parameter to determine the pollution effects of organic wastes. The values for the oxygen dissolved in any sample can not be less than 6 mg·L${}^{-1}$ according to CONAMA [3]. In relation to pH, large variations may occur in this index in case of effluents coming from a domestic or industrial origin. According to Libanio [8] some aquatic organisms such as phytoplankton and zooplankton are usually adapted to the conditions in which the pH is neutral, and abrupt changes in the pH of the water can be harmful to such bodies. Values between 6 and 9 for human drinkable water in Brazil are acceptable according to CONAMA [3].

Another variable of interest on the water analysis process is the turbidity. In water bodies, for example, light penetration is reduced due to turbidity, also reducing the process of photosynthesis [9]. In the case of drinkable water, the turbidity desirable value is of up to 5 nefelometric unities, and up to 40 unities for river and lakes water [10]. Water in its pure state has the capability of solubilizing substances, particularly salts, thus causing some natural waters to having great values of electric conductivity. This conductivity somewhat depends on the stoichiometry of the dissolved mineral (anions or cations) and on its concentration [11]. Conductivity also increases with temperature [8]. It is expressed in microSiemens per centimeter (μS/cm) presenting similar characteristics to the dissolved total solids. In natural waters they can be present values below 100 μS/cm, and reaching up to 1000 μS/cm when the water receives domestic and industrial effluents [7,8].

Temperature is directly linked to the amount of dissolved oxygen. It may influence biological, chemical, and biochemical reactions and the solubility of dissolved gas. This parameter is important for biological productivity, the main limiting factor in the geographical distribution of many species of plants and animals [12]. The water can be classified as cold when the average annual temperature is 19 ${}^{\circ }$C or lower. Brazilian laws do not establish a maximum temperature for water. Canadian and American standards stipulate a maximum value of 15 ${}^{\circ }$C [8]. The redox potential (ORP—Oxidation-Reduction Potential) measures the environmental capability of providing electrons to an oxidizing agent, or of removing electrons from a reducing agent, thus characterizing the oxidation-reduction state in the water. The redox potential is usually expressed in mV [7]. Moreover, from the ORP values, it is possible to analyze the existence of metals such as iron and manganese [13].

In order to provide data of interest in the process of water quality analysis and also to validate the proposed system, the variables addressed in this work are: pH, conductivity, temperature, concentration of dissolved $O_{2}$, and ORP. It is worth mentioning that it is possible to increase the amount of variables for each acquisition module, as is explained later in this article the system is scalable to up to 16. As for example, the nitrite and nitrate that are important in the process of identification of marine life could be used. So, depending on the analysis one wants to do, other variables can be measured by simply inserting the sensor necessary for making certain measurement in the system and giving support to this measurement in telemetry. However, the previously presented parameters are quite found from applications with a high degree of complexity to simple applications and enough to validate the system architecture.

### 2.2. Sensing the Variables in Water

**Figure 1:**
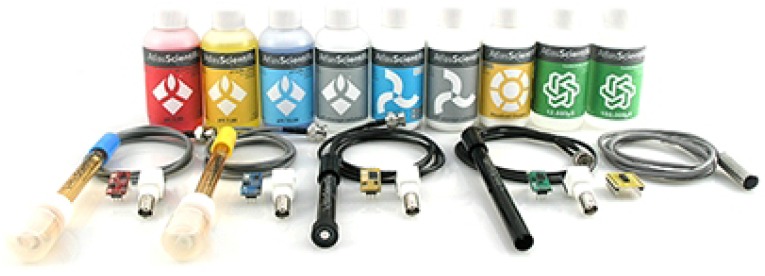
Kit of sensors adopted for water monitoring.

For each variable, a sensor is used to capture the corresponding data. For understanding such system, some details of the sensors used along this research are presented. Due to the simplicity of its use, its direct application as an embedded system, and its low cost, the Atlas Scientific kit shown in Figure 1 [14] is the sensing platform chosen to be used in this work. We remark that this is a commercially available kit that can be adapted to any system, thus needing of further developments both in hardware and software in order to be finally operating. In fact, we could see this as an advantage as one can develop applications in different ways, using faster and cheaper embedded computers in these applications. So, at the cost of more development one can have a better sensing platform.

This kit has sensors for measuring pH, conductivity, temperature, concentration of $O_{2}$, and ORP, as shown in Figure 1. The cost of the kit that comes with some chemical products needed for calibration is $713.95. For comparison of the listed price, a kit of commercial sensors from AGSolve, a company specialized in sensing and monitoring with sensors for the same variables mentioned above, adding depth and a device used to calibrate and visualize data, costs, on average, some three to four times more.

Besides the low cost, another good characteristic of this sensor kit is the interface for its implementation as an embedded system that makes simpler its use as an installed hardware. It comes with connectors and small EZO^TM^ circuit boards. Basically the board receives the signals from sensors and convert them to the corresponding measure unity of each one of them thus making easy data acquisition. Also, the EZO boards allow one to do sensor calibration, storing compensation information at their internal EEPROM (Electrically-Erasable Programmable Read-Only Memory), and to communicate with microcontrollers and/or microprocessors through the protocols *UART* and *I^2^C*.

### 2.3. Using a Sailboat Robot for Water Monitoring

A sensing platform for measuring water quality could be implemented in different ways, for example as several sensors put on vessels or on buoys at some fixed points in some part of a body of water, or even inside an UAV (underwater unmanned vehicle). The sensors platform proposed here is designed to work installed on a surface unmanned vehicle (SUV), named N-Boat—The Sailboat Robot [4]. This sailboat is autonomous, in constant motion, eventually, in the moment of a sensors reading. Thus we impose a restriction for the kit that should not be always in touch with the water, but occasionally on set points. With this, the boat has a better maneuvering. Also, because the sensors are not all the time in the water they could have a longer operational life avoiding accumulation of algae on their surfaces and/or avoiding breaking in some obstacle or so. So the platform is designed to enter the water and to leave it in the next one or two minutes as the sensors have reported the measures to the embedded system that is also on-board working. In summary, in this work we decided to join this with another project that is currently going on, the development of a robotic sailboat, coming up with a complete, effective, and autonomous operating platform for measuring water quality. Note that the above definition of monitoring does not mean constantly taking measures. Instead, it is supposed to be a long endurance mission in which measures on the same points can be taken repeatedly, but respecting some minimum and maximum time from the last measure, say at every other day. Note that an autonomous sailboat can be used to do this with only one sensors kit installed on it, passing through predefined points rather than putting lots of fixed vases or buoys.

Another concern is related to minimizing the power consumption, which is a central issue that has being recently addressed in robotics applications as monitoring. In this direction, using an autonomous robotic sailboat seems to be good choice when monitoring large portions of water in a manner that can be efficient. Moving the robot onto the water surface is done by the wind pushing the sails. Some little energy is spent for driving the sails and the rudder to desired positions. This approach is green computing, because it is autonomous in all aspects, energy generated by solar cells, stored in two 105 A batteries, is enough for the sail and rudder motors [4] and for the operation of the sensors, what is quite interesting. In order to better understand the proposal of this work, we start presenting some theory related to the problem of water monitoring including applications based on sailboat. A comparison between some architectures is presented here.

Some few autonomous robotic sailboat projects are roughly described here in order to better understand the applicability of robotic sailboats to monitoring. The FAST project is a robotic sailboat developed at FEUP (Porto) University [15] especially for the Microtransat challenge [16] and has as its main characteristic the use of FPGA for its digital control system. The AVALON [17] robotic sailboat has its control based on fuzzy logic running in an embedded computer, being precise, fast and with good maneuverability onto water surface. The ROBOAT [18] developed by researchers at INNOC has application similar to our work, in environmental monitoring. It has a simpler structure however with a control system that allows autonommy in trajectory navigation.

**Figure 2:**
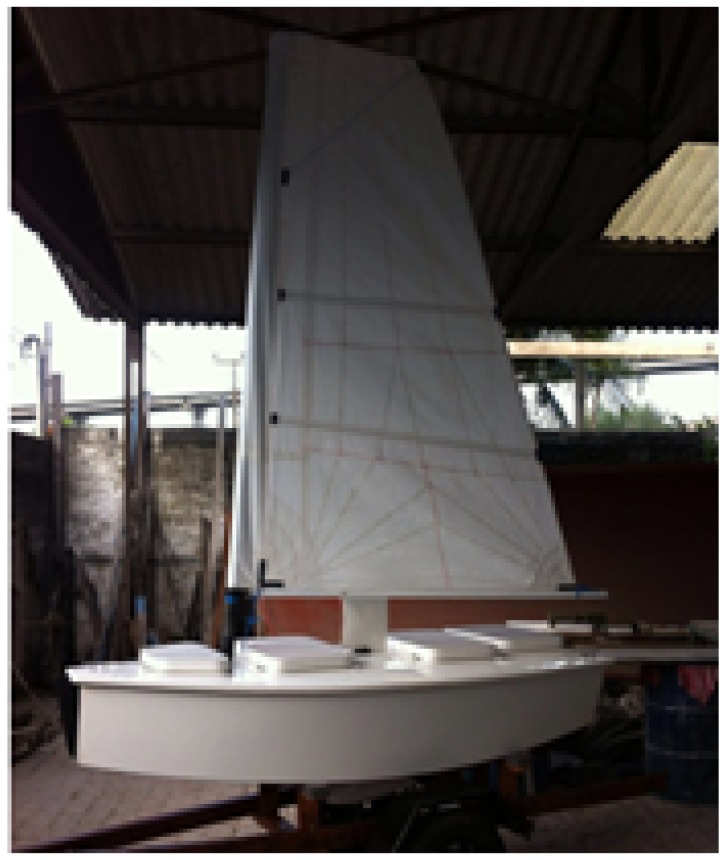
*N-Boat—The Sailboat Robot* construction is currently finalized.

The N-Boat—The Sailboat Robot project [4] is developed by the Laboratory for Perceptual Robotics of Natalnet Associate Labs of UFRN. This sailboat is projected for monitoring of lakes and rivers besides the intention of eventually be competing in the Microtransat challenge. It has a robust control system tested in previous works [4,19] and is planned to have energy sufficiency based on the use of solar panels for charging the two 105 A nautical batteries. It is developed for autonomous navigation thus being ideal for long endurance monitoring. The first version of N-Boat is actually fully working after integration of the several works developed. Currently a new version of N-Boat with 2.4 m long (twice the size of the first version) is finalized. The sensors architecture proposed in the current work will operate installed in this new version of N-Boat. Figure 2 shows the N-Boat II already finalized, the hardware and mechanical parts of the sensors platform will be placed at at its bow (in the frontal part of it). The hardware (circuits, embedded computer) and mechanical parts necessary for operating the sensors (motors, gears, spool) will be inside the first waterproof compartment seen in the bow, with only the sensors left outside the boat, at one of its side.

## 3. Related Projects for Water Analysis Systems

In addition to the sailboats presented in Section 2.3, other projects have been developed specifically for water monitoring. Systems known as RTRM (Real Time Remote Monitoring) are interesting solutions for remote acquisition of data for water quality analysis. Last developments on wireless network communication have enabled substantial advances in this field and is fundamental in the design and construction of these monitoring systems [20]. Below we discuss some projects, programs and institutions that study and apply knowledge in the RTRM area focused on analysis of water quality. Some of these are used as basis or inspiration for the current project, as will be discussed later in more details.

The NEMRP (Neuse Estuary Monitoring and Research Program) keeps a network of 18 stations with 4 to 10 autonomous remote monitoring platforms, working in real time, and provides the data to researchers and educational applications. The monitoring stations are placed strategically in the Neuse River. This program provides a web-based system that stores data in databases and makes them available to the community of interest. The DNREC (Delaware Departament of Natural Resources and Environmental Control) has developed in partnership with the University of Delaware (College of Enginnering, Newark, NJ, USA) an RTRM that is a complete platform for water analysis. Similar to NEMRP, their system acquires, analyzes and provides data on water quality at a given point of the reservoir in real time.

The BTM (Bermuda Tested Mooring), funded by the National Science Foundation (NSF), provides oceanographic data since 1994 [21]. Located at 80 km from the coast of Southern Bermuda, this platform collects meteorological and oceanic water quality data. Among these data, we can highlight temperature, current, conductivity, characteristics relative to optical properties, and the amount of nitrate in the water. This project can be compared to weather buoys widely used by research institutes in time forecasting.

The MARVIN (Merhab Autonomous Research Vessel for In-Situ Sampling) consists basically of a vessel used to real-time monitoring the water quality. This project is the closest one to the proposed architecture in this work, as it also uses a marine vehicle for the same purpose. The vessel, besides allowed to move, does not do it in autonomous way, just the monitoring task itself is autonomous. Uploaded with sensors such as pH, conductivity, turbidity and temperature, Marvin is able to provide real-time data from points of a water body and sends them via satellite through an antenna constructed on the ship [22].

The Mote Marine Laboratory is composed of scientists and sea explorators located at Florida. Their main goal is to monitoring the ocean for guaranteeing sea life. Between their several works, one consists on the development of an underwater autonomous vehicle, also focused in the analysis of water quality. This UAV has been used in research related to phytoplankton and for the mapping of red tides in the Gulf of Mexico. The vehicle is submersible thus allowing to get data from deep water. Data including water variables similar to the above mentioned are made available to Lab users.

The above projects presented in this Section have important features and characteristics that are useful for the foundations of this work. Table 1 summarizes these characteristics. Monitoring refers to long endurance operation in monitoring. For example, some of the platforms are only, occasionally monitoring since the motor boats or submarine present battery costs for the propulsion engines, and can not therefore be used for long periods of time. Autonomous means the monitoring cycle is done in automatic way not needing human interference for analyzing results and displaying graphics and results for the user. Embracing means the developed system is able to monitor (embrace) an area and not just an specific point. Scalable means that more sensors can be added to the platform with only a few or even with no further work in hardware development and software implementation. Finally, the system operation in real-time is the last desired good characteristic (all of them comply with this one).

Our project has met all the above characteristics, joining the main advantages of using an autonomous robotic sailboat (displacement, wind-propelled, less battery consumption, speed and accuracy), with the concepts of remote monitoring systems for real-time analysis of water, so as to obtain a final automated, robust and complete system. It is possible to allocate a sailboat for monitoring a pond, for example, in a way completely autonomous, for several days without the need for human intervention. Also, data can be transmitted and made available through the Internet. Currently we are building the final interface with a site that is available for it at, without restriction to of access to any web user, for now. Also, as our system meet all of the desirable characteristics, we can verify that this proposal is at least equals or better than the ones described.

## 4. The Proposed System Architecture

**Figure 3:**
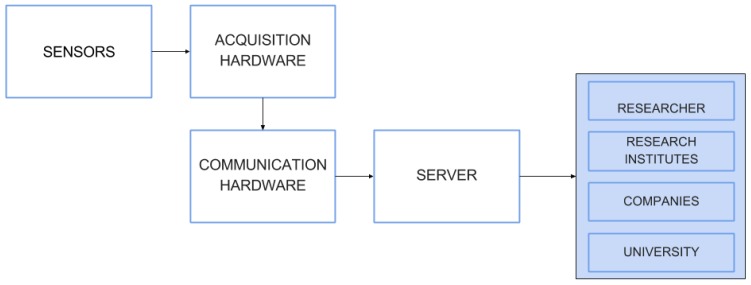
Working flow of the proposed system.

In order for the mechatronic system proposed here to work correctly, we have designed and implemented a hardware architecture taking into account specific needs of the system. As well, a well devised software architecture is necessary to make the system components interacting to each other in a desirable way, in order for the whole system to work as expected. The general operating flow devised for the monitoring system is ilustrated in Figure 3. Basically, the system acquires data from sensors by way of an acquisition hardware commanded by the Central Computer. This computer receives data through some low-level communication protocol, process them and sends them through another communication protocol to a web server (which is remotely located). This server makes data available to final users by way of using a dynamic web page, which allows the updating of them in real time. These data can be accessed on the Internet (with a prototype page working at by any (further registered, if necessary) users. Both hardware and system architectures are detailed in the following.

### 4.1. Hardware Architecture of the System

**Figure 4:**
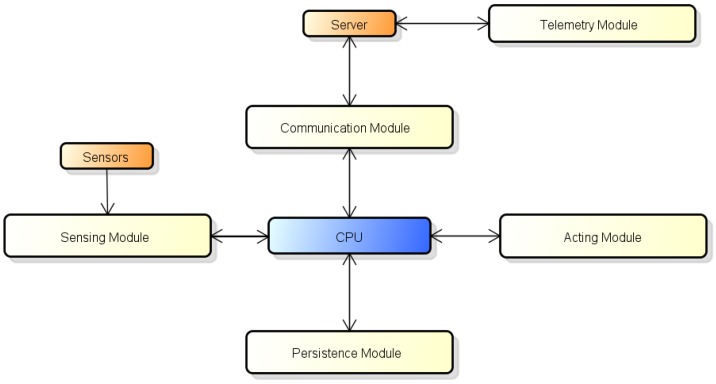
High-level hardware architecture.

The hardware architecture is divided in 5 modules: Sensor, Communication, Persistency, Actuator, and Telemetry. Besides these, the system has a Central Computer, which is a single board computer (SBC) for the processing control, and a (remote) Web Server that is responsible for making data available to users. Figure 4 shows the details of the proposed high-level hardware architecture.

The Central Processor (or processing unity) used is a Raspberry Pi (RPi) model B. This model has 256 Mb of RAM memory and a 700 MHz processor and its architecture allows the development of several, different embedded system projects through the GPIO pins (there are 26 pins in this version). This makes easier the interaction with other hardware. The RPi is a very low-cost computer, with the size of a credit card, which supports Unix platforms. The RPi has support to the SPI communication through pins: SCLK (pin 23), MISO (pin 21), MOSE (pin 19), and SS (pin 24). In the proposed architecture, the RPi is the master of the SPI communication protocol and receives sensor data from the data acquisition module, processes them and sends them to the communication module.

Nonetheless, beyond the above cited features, there are other reasons for choosing Raspberry Pi. One main reason is that it allows communication with the sensors. This could be via SPI (as is the case with the eight ports available for the A/D converter MCP3008) or via I2c, such as in the Atlas Scientific sensors. As a plus, it makes communication easier with the sailboat (the system is applied to the N-Boat robot), which carries an internal server thus it has the option of accessing directly this server system instead of having an external server, and also it is able to store data (MySql database). If we would use another processing unit, for example a dedicated micro-controller, much more things would be needed like storage Shields and communication, between others. With this in mind, we have chosen the Raspberry Pi for being the central computer of the monitoring system, which has made easier our work due to the above features.

The Sensor module is used to acquire sensors data and to send them to the processing unity. Up to eight analog sensors can be used besides the default sensors of the Atlas Scientific kit that are used here to monitor water quality. The components of this module are the sensors themselves, plus the ADC MCP3008 that is used to communicate with the RPi via the SPI protocol. Basically, the MISO and MOSI pins can be used to send and receive data; the clock synchronizes communication; and the SS pin is used by the master to select which slave is planned to be used for exchanging of messages. The formatting of the messages sent between the master and the slave in SPI protocol is beyond the scope of this article, materials on this communication protocol can be found in related references [23]. The EZO boards that come with the sensors kit communicates directly with the RPi via the I^2^C protocol [23]. We remark that the current system is scalable up to sixteen sensor channels, being eight analog channels and eight digital ones, so that other sensors could be devised and added to it. Actually, as discussed in Section 2, one could add new data acquisition modules, since the communication supports multiple slaves. To do that, each module could be added to the communication bus, corresponding to two new slaves.

**Figure 5:**
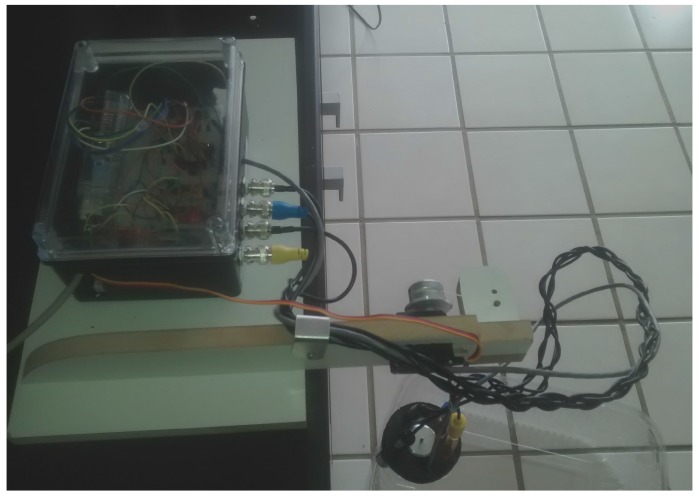
Physical system prototype, in the botton it can be seen a piece of wood with a spool (attached to a servomotor) where it runs the thread that put the sensors in and out of the water; the electronic circuit prototyped in a board can be seen in the top left box (will be seen in detail in Figure 6); the sensors are (waterproof) coupled at the end of the braided cables seen in the right botton.

The Actuator module is responsible for submerging the sensors into the water, and to retrieving them back once the measurements have been done. As discussed above, this strategy is adopted in this work to turn easier the navigation of the sailboat (autonomous) and to avoid problems (caused by alga or breaking) in the sensors in case they are permanently in contact to the water. We remark that the Atlas kit sensors are waterproof and come with cables extensions that are also water resistant. So the probes could be fully submerged in fresh water or in salt water up to the BNC connector indefinitely [14]. So this subsystem, besides hardware connections and the SBC processing, only need to be tightened on a spool of thread and to have a servo motor for leaving it in and out the water. The programming steps for sample acquisition is quite simple. The central computer needs to send commands for the actuator to put the sensors into the water in some specific points as defined by some high-level mission planner and waiting some time (less than two minutes) while a reading is performed. Note that by using this actuator, the system can even acquire measures at different depths, it only needs to send commands some time after a data acquisition is performed, for the actuator to move the sensors to another desired depth. After all samples are acquired at a given place, the sensors can be removed from the water and the sailboat navigate to another predefined way-point. The Actuator module (hardware) is basically composed by an AtMega micro-controller that communicates with the central computer using the I^2^C protocol plus the constructed platform for supporting the kit and spool seen in bottom of Figure 5. Circuit boards necessary for operation of the robotic sailboat and other circuits are not shown here as well as for the power system that allows long endurance operation with autonomy (they are subject of another article [4]).

The communication module is responsible for making data available to the web server. Currently, the system can use Wifi technology or 3G. Some strategies can be devised, such as getting the first (Wifi) in operation in case a routed Internet network appears near the site; the second would start in case the first one does not find the network or fails. Actually, with the further intention of creation of an intranet for remote access, a Wifi router is being used in order to transmit and receive data from the server.

The Persistency module consists of a layer that already exists in the Central computer and in an external server that uses MySQL language in order to store data. It guarantees that data are not lost, persisting stored for some period of time. Besides, this module makes it possible for an user to visualize, on-line, the actual data that are shown in form of graphics in real-time. This module is of fundamental importance in the case of an eventual problem with connection.

Finally, the Telemmetry module performs communication of the system with data consumers of the monitored data. Currently, the components of this module are the Web Server that is also part of the Communication module and the Web Interface itself. The Web Server is implemented as Java servlets running both in the Raspberry Pi and in the remote server. This Web Server allows exchange of data with the RPi with sockets. It is possible to receive data from sensors that are acquired in the Sensor module and to send them, by running AJAX requisitions through a dynamic JSP page. This JSP page presents data from the sensors in real time displaying them in form of graphics that get updated automatically as new data are received.

Further, the system allows to verify the water parameters quality in real time, based on rules predefined by some water managing agency. For example, in Brazil, according to CONAMA [3], the value of the pH for drinkable water shall be in between 6 and 9. Using this predefined information, the system verifies the data and displays in the screen whether the results are inside these predefined bounds.

**Figure 6:**
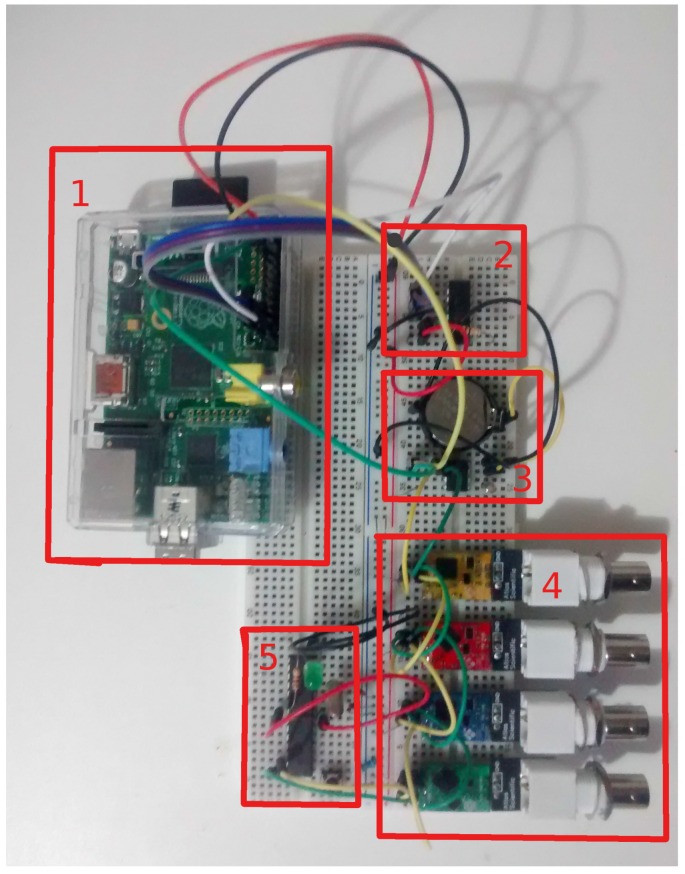
Prototype board of the electronic circuit implemented for the monitoring cycle, the roles/components of each functional part are: (1) Central Computer (RPi); (2) Sensor Module (MCP3008 circuit); (3) Central Computer (RTC circuit); (4) Sensor Module (EZO boards); (5) Actuator Module (AtMega circuit).

**Figure 7:**
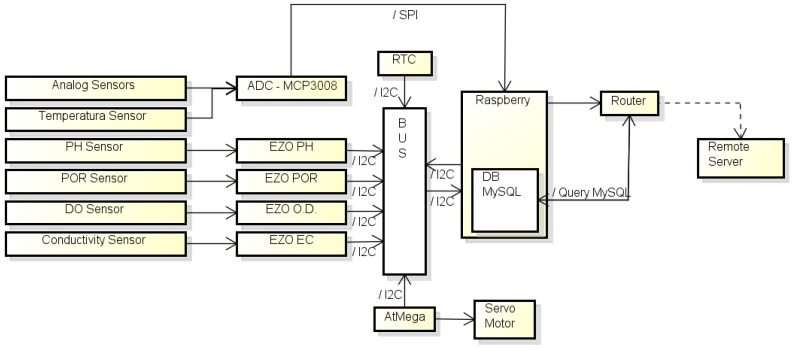
System architecture with used communication protocols.

Figure 7 shows the flow diagram of the system as well the corresponding protocols (or languages) for the components communication. We remark that the electronic circuit board that implements the water monitoring cycle is currently working as a prototype board (to be printed). It can be seen in Figure 6, as well the roles and/or components of each subsystem.

### 4.2. Software Architecture

Two main programs (or processes) are necessary in the system. The first is the embedded process that runs in the RPi, which is responsible for reading data from the sensors, processing them if necessary, and sending them to the remote server. The second program is the process that runs in the server side. It receives data sent by the other process and manages the requisitions coming from the client interface.

#### 4.2.1. Process Embedded in the RPi System

**Figure 8:**
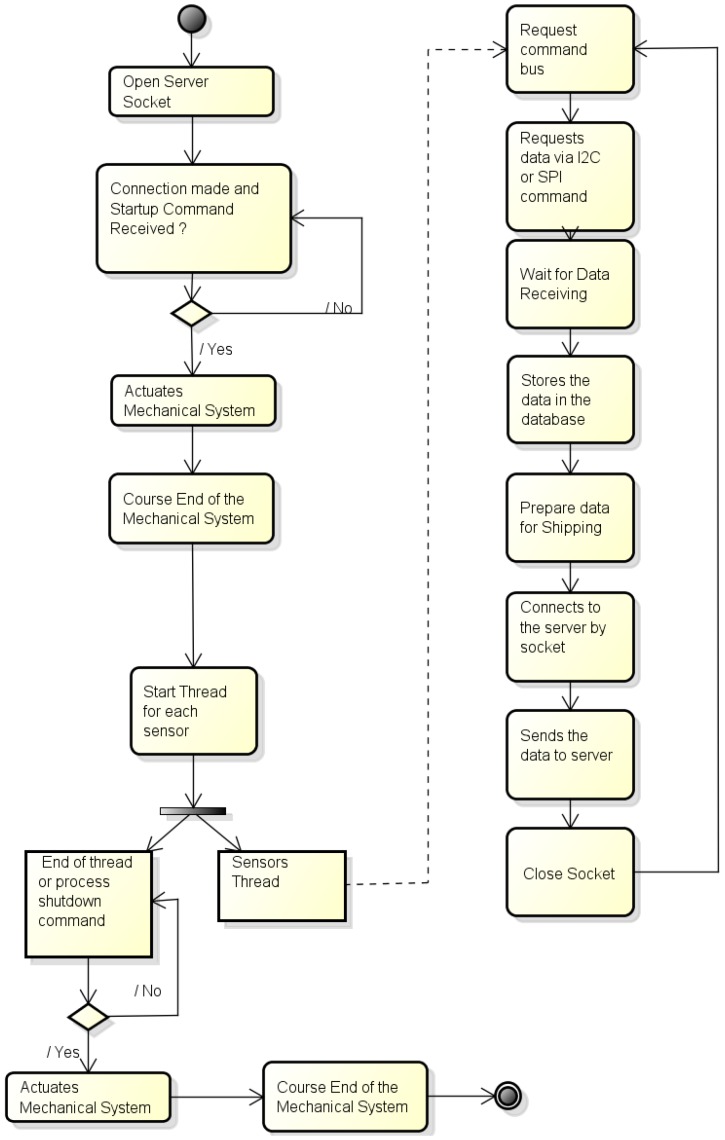
Algorithm of the process running in the Raspberry Pi.

Figure 8 illustrates the flow diagram for the program running in the RPi system. This process should be able to receive, to process, and to send data from the sensors without interference in the system real-time processing. Thus, we have designed an architecture that makes it possible to the system to work adapting it to some sensors characteristics, as for example the need of a one second waiting time for each answer of the EZO board. Besides, this process should also be able to activate the mechanical system (Actuator module) inserting (and removing) the sensors into (and from) the water.

When this process is initiated, a socket server is opened waiting a connection from the client and the system initialization command. When this command arrives, the mechanical system is activated making the sensors submerge into the water. When the mechanical system ends this operation, the data acquisition thread is started. The main program keeps waiting until this thread ends up or else some end command is sent from the system (in case of problems). After data acquisition, they are stored in the database and sent via socket to the web server. This socket is closed and the process restarts until a stop is forced.

#### 4.2.2. Process Running on the Server Side

**Figure 9:**
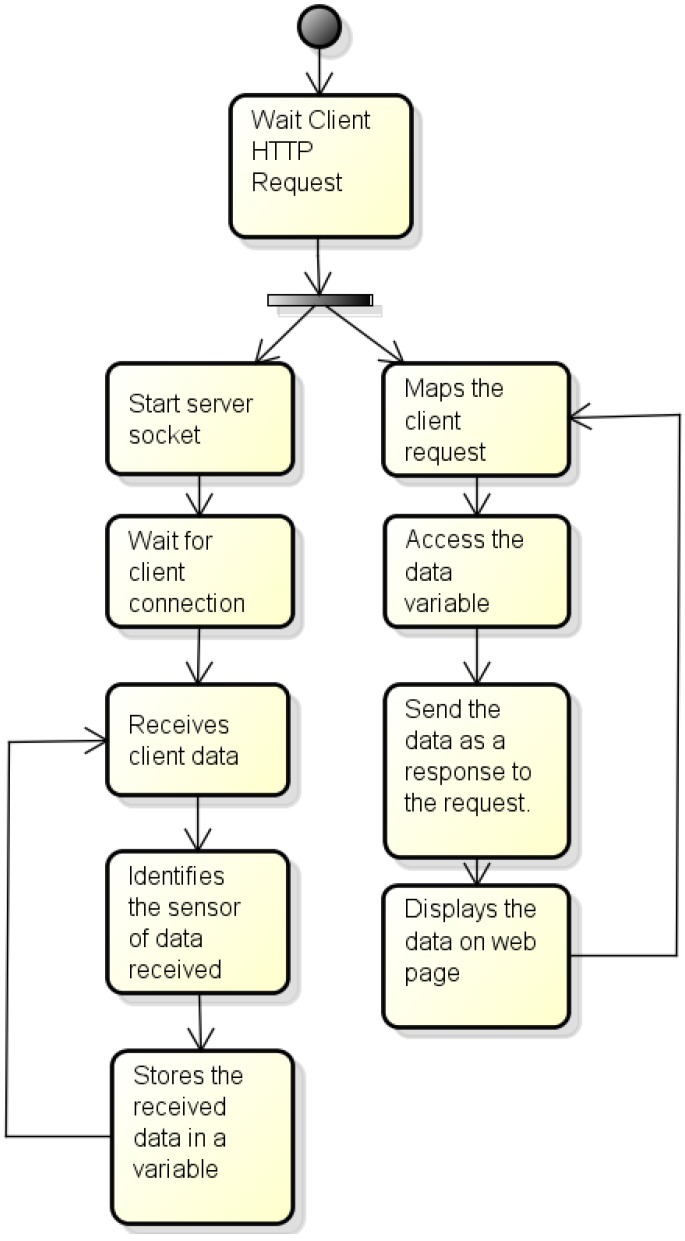
Algorithm of the server process.

The process running on the server side needs to receive data sent from the process embedded in the RPi system, to identify from which sensors these data are coming from, and to store them in local variables in order for them to be further sent to the web server. At the same time, this process should run the connections from the client, sending required data and presenting them in the screen. A working flow diagram for this process is shown in Figure 9.

To this end, through the development of these programs and/or processes and hardware implementations, it was possible to develop the robust system presented in Figure 5. This system is able to perform the complete cycle for monitoring physical-chemical variables of the water, including communication with a web server in order to make available the acquired data to interested people. This proposed system is verified to work in practice as detailed next.

## 5. Experimental Results

With the system prototype working, some experiments are performed in order to verify its correct functioning. A proof of concept and its application for monitoring water quality is necessary. For that, we collected five samples of water data in four different natural resources of water in the northeast of Brazil, two sand dunes natural lakes and two rivers:

- Pium River (Rio Pium, native name in Portuguese);
- Alcaçuz Lake (Lagoa de Alcaçuz, in Portuguese);
- Small River (in fact a river head close to Alcaçuz Lake);
- Blue Lake (Lago Azul, in Portuguese).

**Figure 10:**
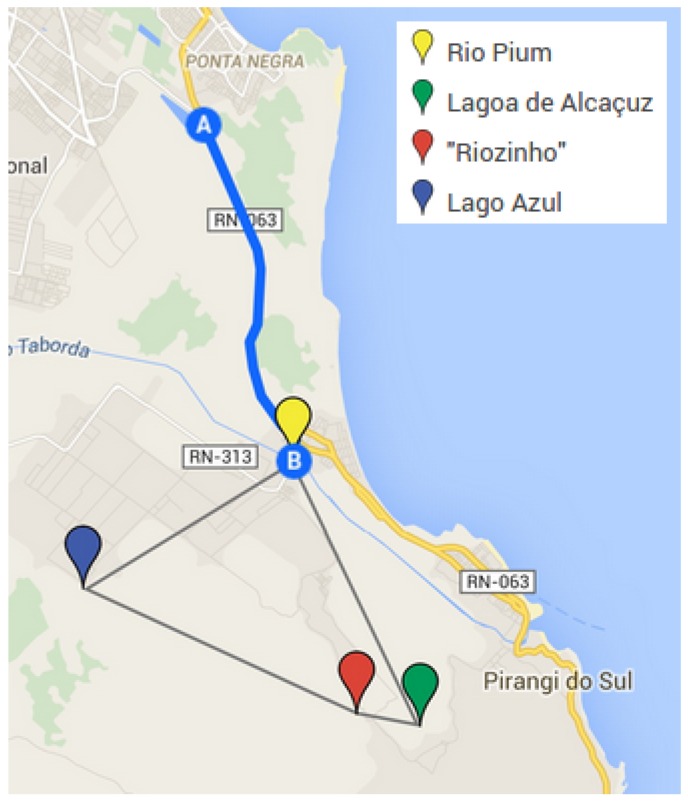
Places for data collection.

Figure 10 shows the places where data are collected. Point A in the map indicates Natal, state capital of Rio Grande do Norte. All the samples were collected in the same day, the N-Boat sailboat was not ready so we brought the system at hand using a vehicle for that and the actuator is put onto a wood platform to acquire data.

**Figure 11:**
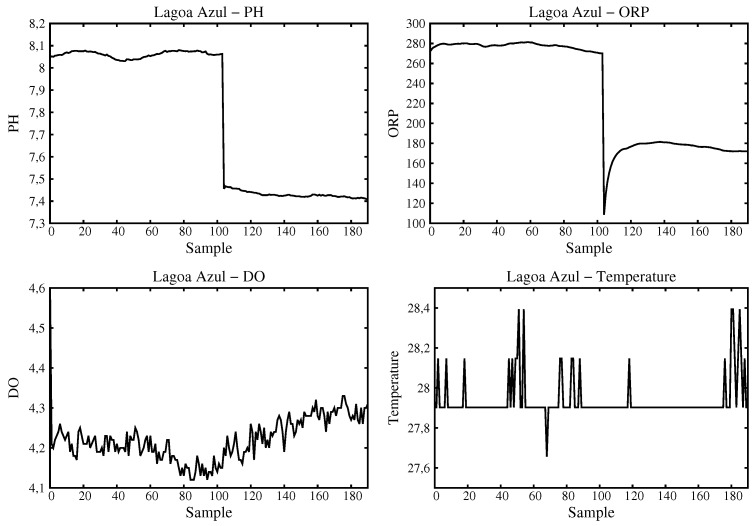
Data acquired and processed for Blue Lake.

We remark that two of the samples were acquired at the Small River, one in its actual source, and the other one a little down, some 500 m away. This second place suffers anthropic interferences caused by people that comes using Buggies, Jeeps and Trollers, that are cars adapted to all terrain, thus supposably modifying the physical-chemical parameters of the water. Figure 11 shows the graphics obtained for the Blue Lake. It is important to note some situations that can be deduced from the analysis of these graphics. For example, the pH as well as the ORP show a subtle variation close to the sample 100. That happens because the sensors were inserted into the water justly after the system becomes up. The oxygen and temperature graphics do not vary considerably.

**Figure 12:**
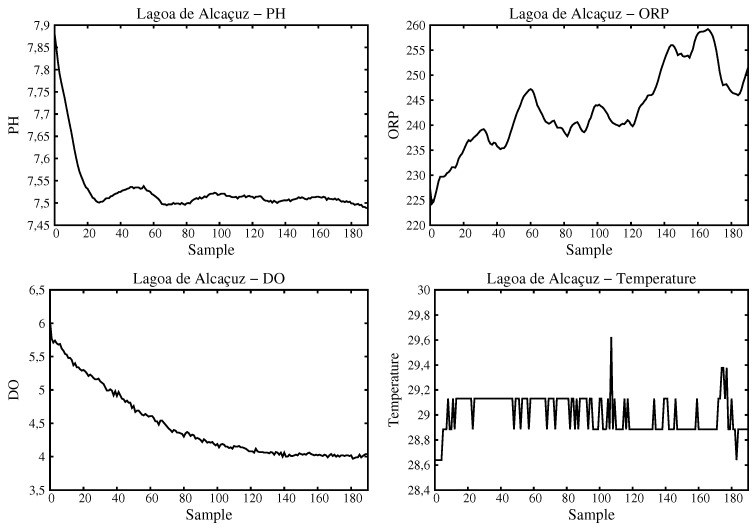
Values of data processed for Alcaçuz Lake.

Figure 12 shows the values obtained for Alcaçuz Lake. As seen, as the sensor was used accordingly, there are no subtle variations if compared to the Blue Lake. The biggest variation has occurred with ORP index that could not stabilize completely going to levels as close to 250 mV.

**Figure 13:**
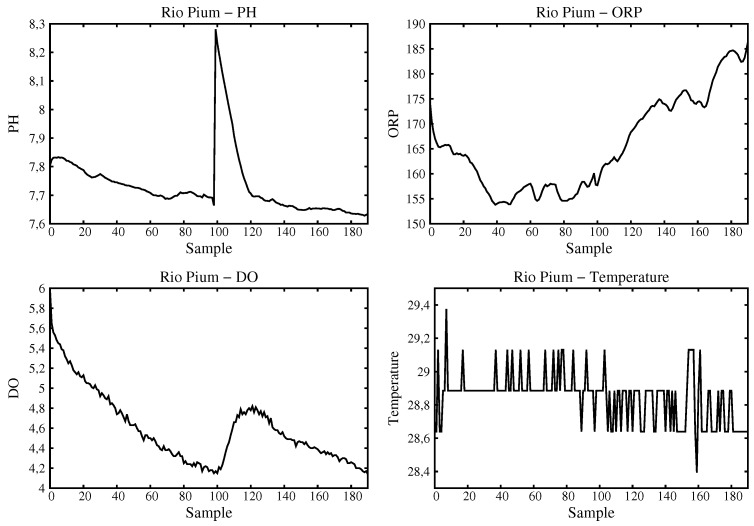
Data acquired and processed for Pium River.

The graphics for data obtained at Pium River are shown in Figure 13. An interesting analysis is the small variation observed for the dissolved oxygen that occurred in between samples 100 and 160. This shows that a unique sampling analysis (as is the case with manual sampling for example) could mislead to wrong values. In this case, as the sensor is in the water for some time, the actual value can be obtained.

**Figure 14:**
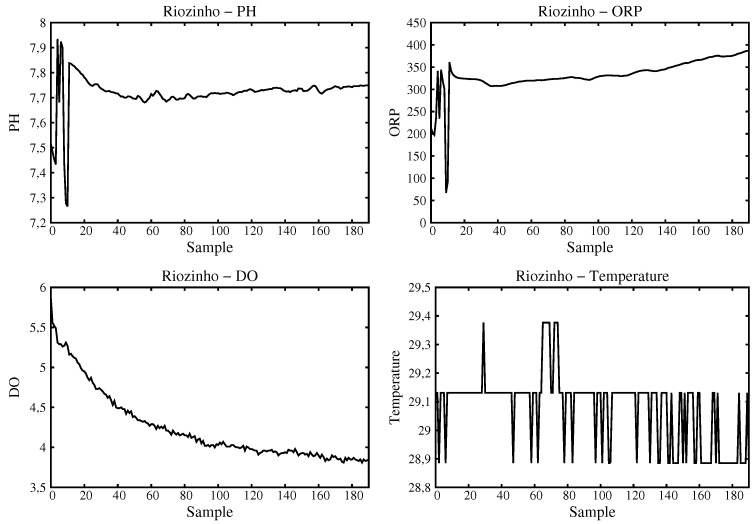
Data acquired and processed for Small River head (a very clean natural source).

**Figure 15:**
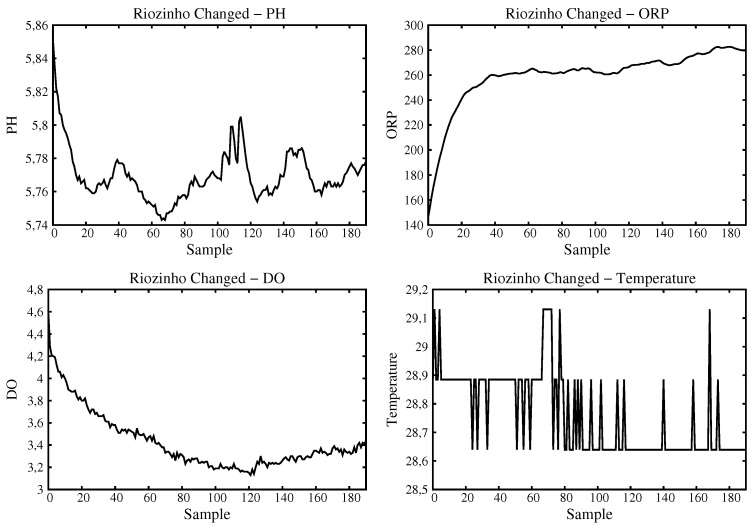
Data acquired and processed for Small River at a place with anthropic actions.

For the Small River, two data samples were acquired. The first at its head (Figure 14) and the second at a place a little down (some 500 m) where it suffers from anthropic actions. There is a passage where owners of off road cars (Buggies, Trollers, and Jeeps) usually like to doing off-road maneuvering as seen in Figure 15. The sample at Small River source has pH values that are neutral and dissolved oxygen close to 4 mg/L, ORP close to 350 mV and temperature stable and close to 29 ${}^{\circ }$C. The down Small River sample shows distorted values. For example, the pH level is modified to values less than 6 meaning that this is not drinkable water. Also, a decrease in the dissolved oxygen value takes place.

### 5.1. The Web Interface

**Figure 16:**
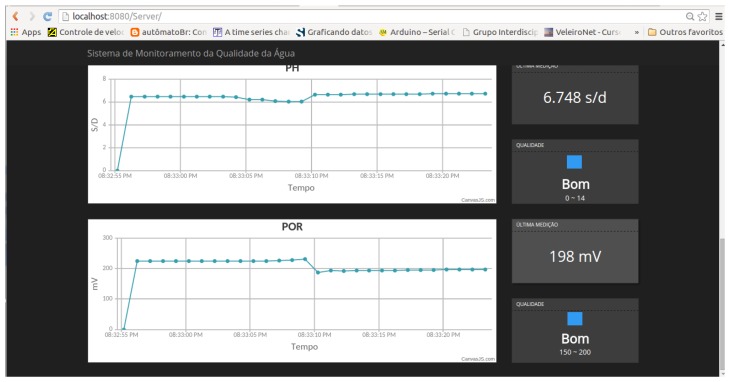
The web interface implemented for showing data on the Internet (already running at www.natalnet.br/nboat).

The web interface can be seen in Figure 16 showing the graphics in the left side. The current measured values of data and the water quality are in the right side. This interface is currently up and running in the Internet at the Natalnet Lab [24] and can be linked to receive data from the the N-Boat throughout several ways, and using other communication devices as a satellite phone, radio link, 3 G and 4 G once the sailboat will operate far away from the base station. We currently made tests using the Digi Xbee-PRO 900HP—Connector RPSMA—Extended-Range that is fully working.

As a way to initially verifying the correct working of the sensors (not a matter for consideration in this paper), we made some manual measures in the above areas only for pH and temperature, while the above samples were acquired by the sensors. In this case, we used a traditional thermometer used in biology classes and a kit used for measuring the pH, and other variables not part of the Atlas kit. In this manually operated measures, the steps are to put a small strip into the water by exactly one minute then taking it out and comparing the color of the strip with some colors that are present in a colortable. The color that is most approximate (visually) to the color given by the strip gives the value interval (say the pH is in between 7.0 and 7.5 for color yellow). We found the values not so precise, as shown in Table 2 for temperature and pH on the measured places. The assertion of ranges values for the pH are due to the impossibility of determining the exact color, instead a range of colors was observed for all measures, that were pretty sure in between two bounds. The values were all between 7 and 7.5 for the pH. We note that the measured values by the sensors were in the range, thus verifying to us that the system works close to the ones given by the manual readings for pH. In respect to the temperature, an error of about 0.2 is verified with the manual registered temperature, which verifies a correct working. We remark that we used these manual experiments only to verify that these two sensors are correctly working, even though this is not a central matter in this paper (just to test to see if the Atlas kit works well).

It is worth noting that the water quality is defined based on parameters given by water managing organisms, national and international. In the case of Brazil, the CONAMA defines these parameters. As for example, the pH in between 6 and 9 that we found in the samples, though one of them was supposedly taken from a dirty place, says that this is drinkable water. Of course this is only with respect to the pH parameter. The other variables also have met these standards for confirming that this is really a drinkable water. In fact, most part of people that live close to those natural resources usually drink water from them or else use the same water for cooking.

## 6. Conclusions

Looking at previous research and at the norms and documents defined by international and national organizations for water monitoring, and motivated by the necessity of a more effective visualization of the water resources in the northeast of Brazil, the architecture of a real-time embedded system for water monitoring is proposed and a prototype is currently implemented to work installed in a sailboat robot. The physical-chemical water parameters that were not so easy to get manually, can now be found on the Internet. As well, buoys can be used to support this system in a fixed model, in this case needing more devices for having a sensors network covering the whole water body.

By analyzing the experiments and results, we conclude that the system is effective, allowing managing organizations to get an idea about the state of the water of some lake (or even the sea), and to take right actions as desired. Also, changes in the behavior of a given body can be perceived in real-time, on-line, allowing mitigation of it in a fast manner.

To this end, through this platform, it is possible to perform the visualization of hydric resources in a better way, by industries, by hydroelectric plants, and also, why not, by the population, in such a way that the governing organizations can be activated in order to take actions against destruction or other problems with the water resources. Corroborating with the 2070 Letter [25], the keeping of life in Earth depends on the efforts done towards preservation of water. This is not the first step towards this direction by our group, however, it is one of the most important in pursuing this goal.

## Figures and Tables

**Table 1:** Comparison of the proposed system with state of art systems.

Projects	Monitoring	Autonomous	Embrancing	Scalabe	Real Time
NEMRP	YES	YES	NO	NO	YES
DNREC	YES	YES	NO	NO	YES
BTM	YES	YES	NO	NO	YES
Marvin	YES	YES	YES	NO	YES
Mote Marine Laboratory	YES	YES	YES	NO	YES
Proposed System	YES	YES	YES	YES	YES

**Table 2:** Comparison betweeen manual manual and sensors kit measures.

Place	pH (Sensor)	pH (Manual)	Temperature (Sensor)	Temperature (Manual)
Pium River	7.7	7 to 7.75	28.9	29
Alcaçus River	7.5	7 to 7.75	29.2	29
Blue Lake	7.45	7 to 7.75	27.8	28
Small River	7.7	7 to 7.75	29.1	29
